# Universal features in the genome-level evolution of protein domains

**DOI:** 10.1186/gb-2009-10-1-r12

**Published:** 2009-01-30

**Authors:** Marco Cosentino Lagomarsino, Alessandro L Sellerio, Philip D Heijning, Bruno Bassetti

**Affiliations:** 1 Università degli Studi di Milano, Dip. Fisica. Via Celoria 16, 20133 Milano, Italy; 2 INFN, Via Celoria 16, 20133 Milano, Italy

## Abstract

**Background:**

Protein domains can be used to study proteome evolution at a coarse scale. In particular, they are found on genomes with notable statistical distributions. It is known that the distribution of domains with a given topology follows a power law. We focus on a further aspect: these distributions, and the number of distinct topologies, follow collective trends, or scaling laws, depending on the total number of domains only, and not on genome-specific features.

**Results:**

We present a stochastic duplication/innovation model, in the class of the so-called 'Chinese restaurant processes', that explains this observation with two universal parameters, representing a minimal number of domains and the relative weight of innovation to duplication. Furthermore, we study a model variant where new topologies are related to occurrence in genomic data, accounting for fold specificity.

**Conclusions:**

Both models have general quantitative agreement with data from hundreds of genomes, which indicates that the domains of a genome are built with a combination of specificity and robust self-organizing phenomena. The latter are related to the basic evolutionary 'moves' of duplication and innovation, and give rise to the observed scaling laws, *a priori *of the specific evolutionary history of a genome. We interpret this as the concurrent effect of neutral and selective drives, which increase duplication and decrease innovation in larger and more complex genomes. The validity of our model would imply that the empirical observation of a small number of folds in nature may be a consequence of their evolution.

## Abstract 2

Novel protein domain stochastic duplication/innovation models that are independent of genome-specific features are used to interpret global trends of genome evolution.

## Background

The availability of many genome sequences provides us with abundant information, which is, however, very difficult to understand. As a consequence, it becomes very important to develop higher-level descriptions of the contents of a genome, in order to advance our global understanding of biological processes. At the level of the proteome, an effective scale of description is provided by protein domains [1]. Domains are the basic modular topologies of folded proteins [2]. They constitute independent thermodynamically stable structures. The physico-chemical properties of a domain determine a set of potential functions and interactions for the protein that carries it, such as DNA- or protein-binding capability or catalytic sites [1,3]. Therefore, domains underlie many of the known genetic interaction networks. For example, a transcription factor or an interacting pair of proteins need the proper binding domains [4,5], whose binding sites define transcription networks and protein-protein interaction networks, respectively.

Protein domains are related to sets of sequences of the protein-coding part of genomes. Multiple sequences give rise to the same topology, so sequence diversity can be explained as a stochastic walk in the space of possible sequences. However, the choice of a specific sequence in this set might also fine-tune the function, activity and specificity of the inherent physico-chemical properties that characterize a topology [6,7]. The topology of a domain then defines naturally a 'domain class', constituted by all its realizations in the genome, in all the proteins using that given fold to perform some function. The connection between the repertoire of protein functions and the set of domains available to a genome is an open problem. This question is related to the fate of domains in the course of evolution, as a consequence of the dynamics of genome growth (by duplication, mutation, horizontal transfer, gene genesis, and so on), gene loss, and reshuffling (for example, by recombination), under the constraints of selective pressure [3,8]. These drives for combinatorial rearrangement, together with the defining modular property of domains, enable the construction of increasingly complex sets of proteins [9]. In other words, domains are particularly flexible evolutionary building blocks.

In particular, the sequences of two duplicate domains that diverged recently will be very similar, so one can also give a strictly evolutionary definition of protein domains [3] as regions of a protein sequence that are highly conserved. The (interdependent) structural and evolutionary definitions of protein domains given above have been used to produce systematic hierarchical taxonomies of domains that combine information about shapes, functions and sequences [10,11]. Generally, one considers three layers, each of which is a supraclassification of the previous one. At the lowest level, domains are grouped into 'families' on the basis of significant sequence similarity and close relatedness in function and structure. Families whose proteins have low sequence identity but whose structures and functional features suggest a common evolutionary origin are grouped in 'superfamilies'. Finally, domains of superfamilies and families are defined as having a common 'fold' if they share the same major secondary structures in the same arrangement and with the same topological connections.

The large-scale data stemming from this classification effort enable us to tackle the challenge of understanding the functional genomics of protein domains [1,12-14]. In particular, they have been used to evaluate the laws governing the distributions of domains and domain families [8,15-18]. As noted by previous investigators, these laws are notable and have a high degree of universality. We reviewed these observations, performing our own analysis of data on folds and superfamilies from the SUPERFAMILY database [19] (Additional data file 1). Using the total number of domains *n *to measure the size of a genome, we make the following observations, which confirm and extend previous ones (note that *n *increases linearly with the number of proteins and, thus, the two measures of genome size are interchangeable; Figure A2.4 in Additional data file 1).

### Observation 1

**Figure 1:**
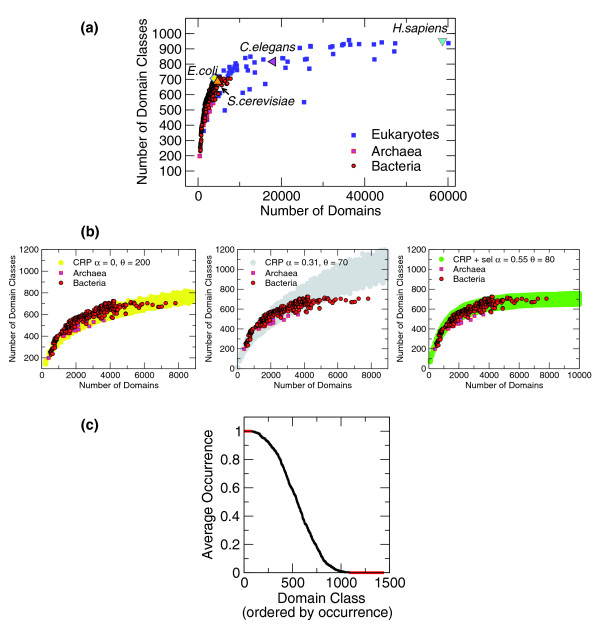
Number of domain classes versus genome size. **(a) **Plot of empirical data for 327 bacteria, 75 eukaryotes, and 27 archaeal genomes. Data refer to superfamily domain classes from the SUPERFAMILY database [19]. Larger data points indicate specific examples. Data on SCOP folds follow the same trend (section A2 in Additional data file 1). **(b) **Comparison of data on prokaryotes (red circles) with simulations of 500 realizations of different variants of the model (yellow, grey, and green shaded areas in the different panels), for fixed parameter values. Data on archaea are shown as squares. *α *= 0 (left panel, graph in log-linear scale) gives a trend that is more compatible with the observed scaling than *α *> 0 (middle panel). However, the empirical distribution of folds in classes is quantitatively more in agreement with *α *> 0 (Table 1 and Figure 2). The model that breaks the symmetry between domain classes and includes specific selection of domain classes (right panel) predicts a saturation of this curve even for high values of *α*, resolving this quantitative conflict. **(c) **Usage profile of SUPERFAMILY domain classes in prokaryotes, used to generate the cost function in the model with specificity. On the x-axis, domain families are ordered by the fraction of genomes they occur in. The y-axis reports their occurrence fraction. The red lines indicate occurrence in all or none of the prokarotic genomes of the data set.

The number of domain classes (or hits of distinct domains) concentrates around a curve *F*(*n*). This means that even genomes that are phylogenetically very distant, but have similar sizes, will have similar numbers of domain classes. This is the case, for example, of the enterobacterium *Shigella flexneris*, with 3,425 domains and 670 distinct domain topologies (giving rise to domain classes), and the distant alkaliphilic Bacillus *Bacillus halodurans*, with 3,406 domains and 637 domain classes. Furthermore, the curve *F*(*n*) is markedly sublinear with size (Figure 1a), perhaps saturating. This means that as the total number of domains *n *measuring genome size expands, the number of different domains becomes strikingly invariant; for example, there is little difference in the number of different domains between *Tetraodon nigroviridis *and *Homo sapiens *despite a doubling in *n*. Interestingly, the same trend is observed within kingdoms, so that, for example, within bacteria both *Escherichia coli *and *Burkholderia xenovorans *(one of the largest bacterial genomes) have 702 distinct domain classes, but n = 3,921 for the former and n = 7,817 for the latter. Note that although the number of domains is increasingly invariant with *n*, the number of proteins is linear in *n*. Hence, the number of different domain combinations in one protein expands, indicating that proteome complexity increasingly relies on combinatorics rather than on number of distinct domain topologies (Figure A2.4 in Additional data file 1).

### Observation 2

**Figure 2:**
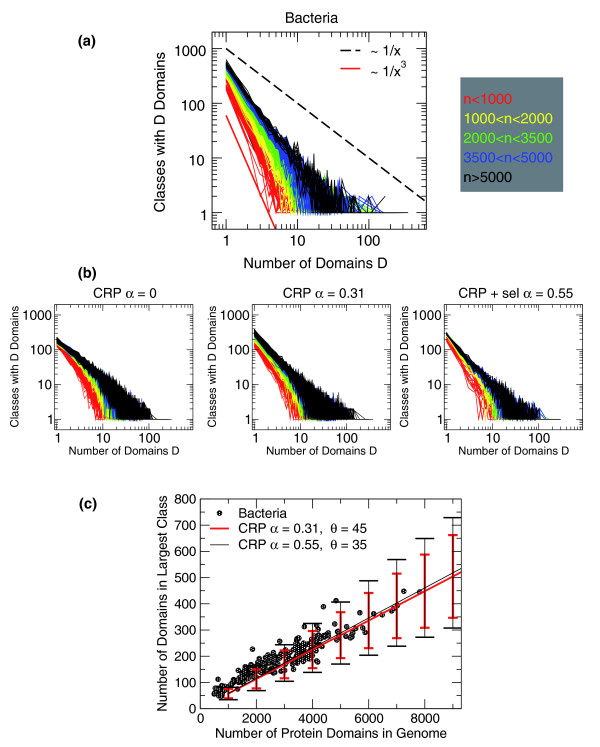
Internal usage of domains. **(a) **Histograms of domain usage; empirical data for 327 bacteria. The x-axis indicates the population of a domain class, and the y-axis reports the number of classes having a given population of domains. Each of the 327 curves is a histogram referring to a different genome. The genome sizes are color-coded as indicated by the legend on the right. Larger genomes (black) tend to have a slower decay, or a larger cutoff, compared to smaller genomes (red). The continuous (red) and dashed (black) lines indicate a decay exponent of 3 and 1, respectively. **(b) **Histograms of domain usage for 50 realizations of the model at genome sizes between 500 and 8,000. The color code is the same as in (a). All data are in qualitative agreement with the empirical data. However, data at *α *= 0 appear to have a faster decay compared to the empirical data. This is also evident looking at the cumulative distributions (section A1 in Additional data file 1). The right panel refers to the model with specificity, at parameter values that reproduce well the empirical number of domain classes at a given genome size (Figure 1). **(c) **Population of the maximally populated domain class as a function of genome size. Empirical data of prokaryotes (green circles) are compared to realizations of the CRP, for two different values of *α*. The lines indicate averages over 500 realizations, with error bars indicating standard deviation. *α *= 0 can reproduce the empirical trend only qualitatively (not shown). Data from the SUPERFAMILY database [19].

The populations of domain classes follow power law distributions. Stated mathematically, the number *F*(*j*,*n*) of domain classes having *j *members (in a genome of size *n*) follows the power law ~ 1/*j*^1+*α*^, where the fitted exponent 1 + *α *typically lies between 1 and 2 (Figure 2). In other words, the population of domain classes tends to have 'hubs' or very populated domain classes. For example, in *E. coli *the hub is the SUPERFAMILY domain 52540 (P-loop containing nucleoside triphosphate hydrolase) with 222 occurrences.

### Observation 3

The slopes tend to become flatter with genome size - that is, the fitted exponent of this power law appears to decrease (Figure 2a) - and there is evidence for a cutoff that increases linearly with *n *(Figure 2c). For example, this cutoff can be measured by the population of the largest class of the hub, and in the case of *B. xenovorans*, the population of the hub is 445, in accordance with the above-mentioned nearly double genome size in terms of domains compared to *E. coli*.

These observed 'scaling laws' are related to the evolution of genomes. In particular, we explore them using abstract models that contain the basic moves available to evolution: domain addition, duplication, and loss. Recent modeling efforts have focused mainly on observation 2, or the fact that the domain class distributions are power laws. They have explored two main directions, a 'designability' hypothesis and a 'genome growth' hypothesis. The designability hypothesis [20] claims that domain occurrence is due to accessibility of shapes in sequence space. While the debate is open, this alone seems to be an insufficient explanation, given, for example, the monophyly of most folds in the taxonomy [3,21]. The 'genome growth' hypothesis, which ascribes the emergence of power laws to a generic preferential-attachment principle due to gene duplication, seems to be more promising. Growth models were formulated as nonstationary, duplication-innovation models [8,22,23], and as stationary birth-death-innovation models [16,24-26]. They were successful in describing to a consistent quantitative extent the observed power laws. However, in both cases, each genome was fitted by the model with a specific set of kinetic coefficients, governing duplication, influx of new domain classes, or death of domains. Another approach used the same modeling principles in terms of a network view of homology relationships within the collective of all protein structures [27,28].

On the other hand, the common trend for the number of domain classes at a given genome size and the common behavior of the observed power laws in different organisms having the same size (observations 1-3), call for a unifying behavior in these distributions, which has not been addressed so far. Here, we define and relate to the data a non-stationary duplication-innovation model in the spirit of Gerstein and coworkers [8]. Compared to this work, our main idea is that a newly added domain class is treated as a dependent random variable, conditioned by the preexisting coding genome structure in terms of domain classes and number. We will show that this model explains the three observations made above with a unique underlying stochastic process having only two universal parameters of simple biological interpretation, the most important of which is related to the relative weight of adding a domain belonging to a new family and duplicating an existing one. In order to reproduce the data, the innovation probability of the model has to decrease with proteome size, that is, such as it is less likely to find new domains in genomes with increasingly larger numbers of domains. This feature is absent in previous models, and opens an interesting biological question: why should the a newly added domain be conditioned on pre-existing domain classes and number? The possible explanations for this phenomenon can be neutral, or selective. Neutral explanations are related to the decreasing effective population size with increasing genome size, which would increase the probability of duplication over innovation for larger genomes, or to the effective pool of available domains, which would decrease the probability of innovation. The main selective argument is that a new domain is likely to be favored only if it can perform a task not covered by pre-existing domains or their combinations. Hence, as the number of domains increases, the chance that a new one will be accepted should decrease. Along the same lines, we also suggest the possibility to interpret this trend as a consequence of the computational cost of adding a new domain class in a genome, manifested by an increasing number of copies of old domains, building up new proteins and interactions needed for adding and wiring a new domain shape into the existing regulatory network. The model generalizes to the presence of domain loss, and we have verified that the same results hold in the limiting hypothesis that domain loss is not dominant (that is, genomes are not globally contracting on average). Finally, we show how the specificity of domain shapes, introduced in the model using empirical data on the usage of domain classes across genomes, can improve the quantitative agreement of the model with data, and in particular predict the saturation of the number of domain classes *F*(*n*) at large genome sizes.

## Results

### Main model

#### Ingredients

**Figure 3:**
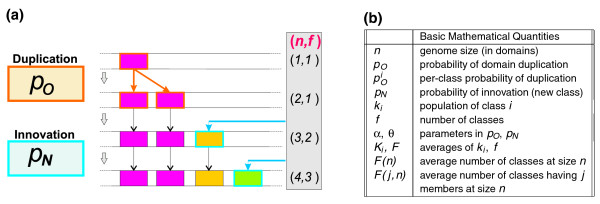
Evolutionary model. **(a) **Scheme of the basic moves. A domain of a given class (represented by its color) is duplicated with probability *p*_*N*_, giving rise to a new member of the same family (hence filled with the same color). Alternatively, an innovation move creates a domain belonging to a new domain class (new color) with probability *p*_*N*_. **(b) **Summary of the main mathematical quantities and parameters of the model.

An illustration of the model and a table outlining the main parameters and observables are presented in Figure 3. The basic ingredients of the model are *p*_*O*_, the probability to duplicate an old domain (modeling gene duplication), and *p*_*N*_, the probability to add a new domain class with one member (which describes domain innovation, for example by horizontal transfer). Iteratively, either a domain is duplicated with the former probability or a new domain class is added with the latter.

An important feature of the duplication move is the (null) hypothesis that duplication of a domain has uniform probability along the genome and, thus, it is more probable to pick a domain of a larger class. This is a common feature with previous models [8]. This hypothesis creates a 'preferential attachment' principle, stating the fact that duplication is more likely in a larger domain class, which, in this model as in previous ones, is responsible for the emergence of power law distributions. In mathematical terms, if the duplication probability is split as the sum of per-class probabilities *p*^*i*^_*O*_, this hypothesis requires that *p*^*i*^_*O*_ ∝ *k*_*i *_, where *k*_*i *_is the population of class *i*, that is, the probability of finding a domain of a particular class and duplicating it is proportional to the number of members of that class.

It is important to note that in this model the relevant parameter is *n*. As pointed out in [8], this parameter is related to evolutionary time in a very complex way, by nonlinear history- and genome-dependent rescalings that are difficult to quantify. On the other hand, the weight ratio of innovation to duplication at a given *n *is more precisely defined (as it can be observed in the data we consider), and is set by the ratio *p*_*N*_/*p*_*O*_. In the model of Gerstein and coworkers [8], both probabilities, and hence their ratio, are constant. In other words, the innovation move is considered to be statistically independent from the genome content. This choice has two problems. First, it cannot give the observed sublinear scaling of *F*(*n*). Indeed, if the probability of adding a new domain is constant with *n*, so will be the rate of addition, implying that this quantity will increase, on average, linearly with genome size. It is fair to say that Gerstein and coworkers do not consider the fact that genomes cluster around a common curve (as shown by the data in Figure 1) and think of each as coming from a stochastic process with genome-specific parameters. Second, their choice of constant *p*_*N *_implies that, for larger genomes, the influx of new domain classes is heavily dominant over the flux of duplicated domains in each old class. This again contradicts the data, where additions of domain classes are rarer with increasing genome size.

#### Defining equations and the Chinese restaurant process

On the contrary, motivated by the sublinear scaling of the number of domain classes (observation 1), we consider that *p*_*N *_is conditioned by genome size. We note that, as observed in [23], constant *p*_*N *_makes sense, thinking that new folds emerge from an internal mutation-like process with constant rate rather than from an external flux. This flux, coming, for example, from horizontal transfer, could be thought of as a rare event with Poisson statistics and characteristic time *τ*, during which the influx of domains is *θτ*. For such a process, it is apparent that *f*(*n*) must have a mean value given by $\sum_{j=1}^{n}\frac{\theta }{\theta +n}$, thus increasing as *θ*log *n*. This scenario is complementary to the one of Gerstein and coworkers because old domain classes limit the universe that new classes can explore.

One can think of intermediate scenarios between the two. The simplest scheme, which turns out to be quite general, implies a dependence of *p*_*N *_by *n *and *f*, where *n *is the size (defined again as the total number of domains) and *f *is the number of domain classes in the genome. Precisely, we consider the expressions:

$$p_{O}^{i}=\frac{k_{i}-\alpha }{n+\theta },$$

and since $p_{O}=\sum_{i}p_{O}^{i}$ (that is, the total probability of duplication must coincide with the sum of *per-class *duplication probabilities):

$$p_{O}=\frac{n-\alpha f}{n+\theta },$$

and

$$p_{N}=\frac{\theta +\alpha f}{n+\theta },$$

where *θ *≥ 0 and *α *∈ [0,1]. Here *θ *is the parameter representing a characteristic size *n *needed for the preferential attachment principle to set in, and defines the behavior of *f*(*n*) for vanishing *n*. *α *is the most important parameter, which sets the scaling of the duplication/innovation ratio (see the second column of Table 1). Intuitively, for small *α *the process slows down the growth of *f *at small values of *n *(necessarily *f *≤ *n *because classes have at least one member), and since *p*_*N *_is asymptotically proportional to the class density *f*/*n*, it is harder to add a new domain class in a larger, or more heavily populated genome. As we will see, this implies *p*_*N*_/*p*_*O *_→ 0 as *n *→ ∞, corresponding to an increasingly subdominant influx of new fold classes at larger sizes. We will show that this choice reproduces the sublinear behavior for the number of classes and the power law distributions described in observations 1-3.

**Table 1:** Salient features of the proposed model in terms of scaling of the number of domain classes, compared to the model of Gerstein and coworkers [8,22]

	*K*_*i*_	$\frac{p_{N}}{p_{O}}$	$\frac{p_{N}}{p_{O}^{i}}$	*F*(*n*)	*F*(*j*, *n*)/*F*(*n*)
*CRPα *= 0	~ *n*	~ *n*^-1^	~ *n*^-1^	~ log (*n*)	$\sim \frac{\theta }{j}$
*CRPα *> 0	~ *n*	~ *n*^*α*-1^	~ *n*^*α*-1^	~ *n*^*α*^	~ *j *^-(1+*α*)^
*Qian* et al.	$\sim n^{p_{O}}$	= *R*	$\sim n^{1-p_{O}}$	~ *n*	~ *j*^-(2+*R*)^

The first three columns indicate the resulting average population of a class *K*_*i*_, and the ratios of the probability to add a new class *p*_*N *_to the total and per-class probabilities of duplication, as a function of genome size *n*. These latter two quantities are asymptotically zero in the CRP, while they are constant or infinite in the model of Gerstein and coworkers. The last two columns indicate the resulting scaling of number of domain classes *F*(*n*) and fraction of classes with *j *domains *F*(*j*, *n*)/*F*(*n*). The results of the CRP agree qualitatively with observations 1-3 in the text.

This kind of model has previously been explored in a different context in the mathematical literature under the name of Pitman-Yor, or the Chinese restaurant process (CRP) [29-32]. In the Chinese restaurant metaphor, domain realizations correspond to customers and tables to domain classes. A domain that is a member of a given class is represented by a customer sitting at the corresponding table. In a duplication event, a new customer is seated at a table with a preferential attachment principle, corresponding to the idea that, with table-sharing, customers may prefer more crowded tables because this could be an indication of better or more food (for domains, this feature enters naturally with the null hypothesis of uniform choice of duplicated domains). In an innovation event, the new customer sits at a new table.

#### Theory and simulation

We investigated this process using analytical asymptotic equations and simulations. The natural random variables involved in the process are *f*, the number of tables or domain classes, *k*_*i *_the population of class *i*, and *n*_*i*_, the size at birth of class *i*. Rigorous results for the probability distribution of the fold usage vector (*k*_1_, ..., *k*_*f*_) confirm the results of our scaling argument. It is important to note that in this stochastic process, large *n *limit values of quantities such as *k*_*i *_and *f *do not converge to numbers, but rather to random variables [29].

Despite of this property, it is possible to understand the scaling of the averages *K*_*i *_and *F *(of *k*_*i *_and *f*, respectively) at large *n*, writing simple 'mean-field' equations in the spirit of statistical physics, for continuous *n*. From the definition of the model, we obtain:

$$\partial_{n}K_{i}(n)=\frac{K_{i}-\alpha }{n+\theta }$$

and

$$\partial_{n}F(n)=\frac{\alpha F(n)+\theta }{n+\theta }$$

These equations have to be solved with initial conditions *K*_*i*_(*n*_*i*_) = 1, and *F*(0) = 1. Hence, for *α *≠ 0:

$$K_{i}(n)=(1-\alpha )\frac{n+\theta }{n_{i}+\theta }+\theta$$

and

$$F(n)=\frac{1}{\alpha }\left[(\alpha +\theta ){\left(\frac{n+\theta }{\theta }\right)}^{\alpha }-\theta \right]\sim n^{\alpha },$$

while, for *α *= 0:

*F*(*n*) = *θ*log (*n *+ *θ*) ~ log (*n*).

These results imply that the expected asymptotic scaling of *F*(*n*) is sublinear, in agreement with observation 1.

The mean-field solution can be used to compute the asymptotics of *P*(*j*,*n*) = *F*(*j*,*n*)/*F*(*n*) [33]. This works as follows. From the solution, *j *> *K*_*i*_(*n*) implies *n*_*i *_> n*, with $n*=\frac{\left(1-\alpha )n-\theta (j-1\right)}{j-\alpha }$, so that the cumulative distribution can be estimated by the ratio of the (average) number of domain classes born before size n* and the number of classes born before size *n*, *P*(*K*_*i*_(*n*) > *j*) = *F*(n*)/*F*(*n*). *P*(*j*, *n*) can be obtained by derivation of this function. For *n*, *j *→ ∞, and *j*/*n *small, we find:

*P*(*j*, *n*) ~ *j*^-(1+*α*)^

for *α *≠ 0, and

$$P(j,n)\sim \frac{\theta }{j}$$

for *α *= 0. The above formulas indicate that the average asymptotic behavior of the distribution of domain class populations is a power law with exponent between 1 and 2, in agreeement with observation 2.

The trend of the model of Gerstein and coworkers can be found for constant *p*_*N*_, *p*_*O *_and gives a linearly increasing *F*(*n*) and a power law distribution with exponent larger than two for the domain classes (hence, in general, not compatible with observations). A comparative scheme of the asymptotic results is presented in Table 1. We also verified that these results are stable for introduction of domain loss and global duplications in the model (section A5 in Additional data file 1). Incidentally, we note also that the 'classic' Barabasi-Albert preferential attachment scheme [33] can be reproduced by a modified model where at each step a new domain family (or new network node) with, on average, *m *members (edges of the node) is introduced, and at the same time *m *domains are duplicated (the edges connecting old nodes to the new node).

Going beyond the mean behavior for large sizes *n*, the probability distributions generated by a CRP contain large finite-size effects that are relevant for the experimental genome sizes. In order to evaluate the behavior and estimate parameter values taking into account stochasticity and the small system sizes, we performed direct numerical simulations of different realizations of the stochastic process (Figures 1b and 2b,c). The simulations allow the measurement of *f*(*n*), and *F*(*j*,*n*) for finite sizes, and, in particular, for values of *n *that are comparable to those of observed genomes. At the scales that are relevant for empirical data, finite-size corrections are substantial. Indeed, the asymptotic behavior is typically reached for sizes of the order of *n *~ 10^6^, where the predictions of the mean-field theory are confirmed.

Comparing the histograms of domain occurrence of model and data, it becomes evident that the intrinsic cutoff set by *n *causes the observed drift in the fitted exponent described in observation 3 and shown in Figure 2a,b. In other words, the observed common behavior of the slopes followed by the distribution of domain class population for genomes of similar sizes can be described as the finite-size effects of a common underlying stochastic process. We measured the cutoff of the distributions as the population of the largest domain class, and verified that both model and data follow a linear scaling (Figure 2c). This can be expected from the above asymptotic equations, since *K*_*i*_(*n*) ~ *n*.

The above results show that the CRP model can reproduce the observed qualitative trends for the domain classes and their distributions for all genomes, with one common set of parameters, for which all random realizations of the model lead to a similar behavior. One further question is how quantitatively close the comparison can be. To answer this question, we compared data for the bacterial data sets and models with different parameters (Figures 1b and 2). Note that data concerning eukaryotes refer to scored sequences for all unique proteins, and thus are affected by a certain amount of double counting because of alternative splicing. For this reason, for the quantitative comparison that follows, we only use the data concerning bacteria. On the other hand, we note that the genomes where domain associations are available for the longest transcripts of each gene, and thus are not affected by double counting, the same qualitative behavior is found (Figure A3.6 in Additional data file 1), indicating that the model should apply also to eukaryotes. Considering the data from bacteria, while the agreement with the model is quite good, it is difficult to decide between a model with *α *= 0 and a model with finite (and definite) *α*: while the slope of *F*(*n*) is more compatible with a model having *α *= 0, the slopes of the internal power law distribution of domain families *P*(*j*,*n*) and their cutoff as a function of *n *is in closer agreement to a CRP with *α *between 0.5 and 0.7 (Figure 1b; sections A1 and A2 in Additional data file 1).

### Domain family identity and model with domain specificity

We have seen that the good agreement between model and data from hundreds of genomes is universal and realization-independent. On the other hand, although one can clearly obtain from the basic model all the qualitative phenomenology, the quantitative agreement is not completely satisfactory, as the qualitative behavior observed in the model for *α *> 0 seems to agree better with observed domain distributions.while observed domain class number better agrees with *α *= 0 (Figures 1 and 2).

We will now show how a simple variant of the model that includes a constraint based on empirically measured usage of individual domain classes can bypass the problem, without upsetting the underlying ideas presented above. Indeed, there exist also specific effects, due to the precise functional significance and interdependence of domain classes. These give rise to correlations and trends that are clearly visible in the data, which we analyze in more detail in a parallel study (manuscript in preparation). Here, we will consider simply the empirical probabilities of usage of domain families for 327 bacterial genomes in the SUPERFAMILY database [19] (Figure 1c). These observables are largely uneven, and functional annotations clearly show the existence of ubiquitous domain classes, which correspond to 'core' or vital functions, and marginal ones, which are used for more specialized or contextual scopes. On biological grounds, this fact is expected to have consequences on the basic probabilities of the model. Indeed, if new domain classes in a genome originate by horizontal transfer or by mutation from prior domains, not all domains are equally likely to appear. Those that are rarer are less likely to be added, because horizontal transfers involving them will be rare, or because the barrier to produce them from their precursors is higher. It is then justified to incorporate these effects into the CRP model.

In order to identify model domain classes with empirical ones, it is necessary to label them. We assign each of the labels a positive or negative weight, according to its empirical frequency measured in Figure 1c. A genome can then be assigned a cost function, according to how much its domain family composition resembles the average one. In other words, the genome receives a positive score for every ubiquitous family it uses, and a negative one for every rare domain family. We then introduce a variant in the basic moves of the model, which can be thought of as a genetic algorithm. This variant proceeds as follows. In a first substep, the CRP model generates a population of candidate genome domain compositions, or virtual moves. Subsequently, a second step discards the moves with higher cost, that is, where specific domain classes are used more differently from the average case. Note that the virtual moves could, in principle, be selected using specific criteria that take into account other observed features of the data than the domain family frequency. The model is described more in detail in section A4 in Additional data file 1. We mainly considered the case with two virtual moves, which is accessible analytically. The analytical study also shows that the only salient effective ingredient for obtaining the correct scaling behavior is the fraction of domain classes with positive or negative cost. Using this fact, this variant of the model can be formulated in a way that does not upset the spirit of our formulation of having few significant control parameters.

In the modified model, not all classes are equal. The cost function introduces a significance to the index of the domain class, or a colored 'tablecloth' to the table of the Chinese restaurant. In other words, while the probability distributions in the model are symmetric by switching of labels in domain classes [31], this clearly cannot be the case for the empirical case, where specific folds fulfill specific biological functions. We use the empirical domain class usage to break the symmetry, and make the model more realistic. Moreover, the labels for domain classes identify them with empirical ones, so that the model can be effectively used as a null model.

Simulations and analytical calculations show that this modified model agrees very well with observed data. Figures 1b and 2b show the comparison of simulations with empirical data. The agreement is quantitative. In particular, the values of *α *that better agree with the empirical behavior of the number of domain classes as a function of domain size *F*(*n*) are also those that generate the best slopes in the internal usage histograms *F*(*j*,*n*). Namely, the best *α *values are between 0.5 and 0.7. Furthermore, the cost function generates a critical value of *n*, above which *F*(*n*), the total number of domain families, becomes flat. This behavior agrees with the empirical data better than the asymptotically growing laws of the standard CRP model. A mean-field calculation of the same style as the one presented above predicts the existence of this plateau (section A4 in Additional data file 1).

## Discussion

The model shows that the observed common features, or scaling laws, in the number and population of domain classes of organisms with similar proteome sizes can be explained by the basic evolutionary moves of innovation and duplication. This behavior can be divided into two distinct universal features. The first is the common scaling with genome size of the power laws representing the population distribution of domain classes in a genome. This was reported early on by Huynen and van Nimwegen [15], but was not considered by previous models. The second feature is the number of domain families versus genome size *F*(*n*), which clearly shows that genomes tend to cluster on a common curve. This fact is remarkable, and extends previous observations. For example, while it is known that generally in bacteria horizontal transfer is more widespread than in eukaryotes, the common behavior of innovation and duplication depending on coding genome size only might be unexpected. The sublinear growth of number of domain families with genome size implies that addition of new domains is conditioned to genome size, and, in particular, that additions are rarer with increasing size.

### Comparison with previous modeling studies

Previous literature on modeling of large-scale domain usage concentrated on reproducing the observed power law behavior and did not consider the above-described common trends. In order to explain these trends, we introduce a size dependency in the ratio of innovation to duplication *p*_*N*_/*p*_*O*_. This feature is absent in the model of Gerstein and coworkers, which is the closest to our formalism. We have shown that this choice is generally due to the fact that *p*_*N *_is conditioned by genome size. Furthermore, we can argue on technical grounds that the choice of having constant *p*_*O *_and *p*_*N *_would be more artificial, as follows. If one had $p_{0}^{i}$ = *k*_*i*_/*n*, the total probability *p*_*O *_would be one, since the total population *n *is the sum of the class populations *k*_*i*_, and there would not be innovation. In order to build up an innovation move, and thus *p*_*N *_> 0, one has to subtract small 'bits' of probability from $p_{0}^{i}$. If *p*_*N *_has to be constant, the necessary choice is to take $p_{0}^{i}$ = *k*_*i*_/*n* - *p*_*N*_/*f*, where *f *is the number of domain classes in the genome. This means that the probability of duplication for a member of one class would be awkwardly dependent on the total number of classes.

Furthermore, we have addressed the role of specificity of domain classes, by considering a second model where each class has a specific identity, given by its empirical occurence in the genomes of the SUPERFAMILY data set. This model, which gives up the complete symmetry of domain classes, has the best quantitative agreement with the data, and is a good candidate for a null model designed for genome-scale studies of protein domains. Obviously, the better performance of this model variant has the cost of introducing extra phenomenological parameters, which, however, are not adjustable, but empirically fixed, since each class has its own value determined by its empirical occurrence. Thus, these extra per-class parameters do not need any estimation as *α *and *θ*. One may suspect that this addition weakens the salient point of having a model with few universal parameters. On the other hand, an effective 'parameter-poor' model can reproduce the main results of the specific model, which just depend on the assumption of the existence of two sets of 'universal' versus 'contextual' domain classes, and can be obtained by adding only one extra relevant parameter, the fraction of universal domains. The detailed weight of each empirical class remains important for the possible use as a null model.

### Role of the common evolutionary history of empirical genomes

It is useful to spend a few words on the role of common ancestry in the observed scaling laws, compared to the model. Clearly, empirical genomes come from intertwined evolutionary paths. The model treated here does not include time in generations, but reproduces sets of 'random' different genomes, parameterized by size *n *using the basic moves of duplication and innovation (and also loss, see below). Genomes from the same realization can be thought of as a trivial phylogenetic tree, where each value of *n *gives a new species. In contrast, independent realizations are completely unrelated.

The scaling laws hold both for each realization and, more importantly, for different realizations, indicating that they are properties that stem from the fact that all branches of phylogenetic trees are built with the same basic moves and not from the fact that branches are intertwined. For example, two completely unrelated realizations will reach similar values of *F *at the same value of *n*. In other words, the predictions of the model are essentially the same for all histories (at fixed parameters), which can be taken as an indication that the basic moves are more important in establishing the observed global trends than the shared evolutionary history. This is confirmed by the data, where phylogenetically extremely distant bacteria with similar sizes have nevertheless very similar numbers and population distributions of domain classes.

While the scaling laws are found independently on the realization of the CRP model, the uneven presence of domain classes can be seen as strongly dependent on common evolutionary history. Averaging over independent realizations, the prediction of the standard model would be that the frequency of occurrence of any domain class would be equal, as no class is assigned a specific label. In the Chinese restaurant metaphor, the customers only choose the tables on the basis of their population, and all the tables are equal for any other feature. However, if one considers a single realization, which is an extreme but comparatively more realistic description of common ancestry, the classes that appear first are obviously more common among the genomes. In particular, in the 'specific' variant of the model, the empirically ubiquitous classes are given a lower cost function, and tend to appear first in all realizations.

This model has full quantitative descriptive value on the available data. Its value is also predictive, as removing a few genomes does not affect its power. However, it can be argued that this predictivity is trivial, as there is little biological interest in knowing that a genome behaves just as all the other ones. More interestingly, the model can be used negatively, to verify whether and to what extent a genome deviates from the expected behavior in its domain class composition and population. In other words, we believe that it could be an interesting tool to use as a null model in evolutionary studies of domains at the genome level.

### Role of domain loss

While domain deletion is a common phenomenon, we have chosen to consider (similarly to Gerstein and coworkers) a basic model including duplication and innovation moves only. Inspection of a variant of the model with domain deletion (section A5 in Additional data file 1) shows that addition of domain loss does not change the basic results. Provided domain loss is not dominant (that is, genome sizes are not globally contracting), the extra parameter of domain deletion only determines a correction to the scaling exponents. Therefore, it can be considered a secondary ingredient to reproduce the scaling laws, and the basic model we consider is sufficient to establish the relevant behavior.

The key limitation in the treatment we have performed is the assumption that gene loss is not dominant. While domain loss has been addressed and measured at large scales [14], no quantitative picture is currently available, and, in particular, it has not been established that domain loss cannot be a dominant process at some evolutionary times or in some sectors of the phylogenetic tree. In these conditions, our model would not be applicable as formulated here.

### Role of 'ORFans'

All sequenced genomes contain a large number of 'ORFan' proteins whose domains are not scored by domain databases because of the total lack or a very limited extent of homologs. If all these domains are thought to give rise to singleton domain classes, the observed scaling laws might be affected. In other words, classes corresponding to 'rare' domain topologies are harder to discover, and thus more likely not to be in the databases. This can create some bias in the data if these 'ORFans' do not behave as the observed domains. Assuming they do not, in order for their domain classes to increase linearly with *n*, they have to be added with constant probability, as in the model of Gerstein and coworkers [8]. The available data allow us to exclude that this holds for the observed domains, so that the only remaining possibility is that, assuming ORFans behave differently from observed domains, the genome is composed of two sets of domain topologies with distinct behavior: observable domains follow our model while ORFans follow the model of Gerstein and coworkers.

### Neutral interpretations for the differential domain innovation to duplication ratio with varying proteome size

The next question worth discussing is the possible biological interpretation of the scaling of innovation to duplication, *p*_*N*_/*p*_*O *_as a function of proteome size *n*. As we have shown, this ratio must scale in the correct way with *n *in order to reproduce the data. As shown in Table 1 and in Figures 1 and 2, this is set by the parameter *α *of the model. Precisely, the ratio *p*_*N*_/*p*_*O *_decreases like ~ *n*^*α*-1^. In other words, necessarily something affects the addition of domains with new structures relative to domains with old structures, making it sparser with increasing size. This fact is not a prediction of the model, but rather a feature of the data, which constrain the model. Note that innovation events can have the three basic interpretations of horizontal transfers carrying new domain classes, gene-genesis or splitting of domain classes when internal structures diverge greatly, while duplication events can be interpreted as real duplication, or horizontal transfers carrying domains that belong to domain classes already present in the genome. While this might be confusing if one focuses on the genome, it seems reasonable to associate these processes to true 'innovations' and 'duplications' at the protein level. At least for bacteria, innovation by horizontal transfer could be the most likely event. In this case, the question could be reduced to asking why the relative rate of horizontal transfer of exogenous domain classes decreases with proteome size relative to the sum of duplication and horizontal transfer of endogenous domain classes.

In order for *p*_*N*_/*p*_*O *_to decrease with *n*, either *p*_*O *_has to increase, or *p*_*N *_has to decrease, or both. A possible source of increase of *p*_0 _with *n *is the effective population size. Recent studies [34] suggest that coding genome size correlates with population size, and in turn this results in reduced selective pressure, allowing the evolution of larger genomes. Thus, one can imagine that the ease to produce new duplications and proteome size are expected to correlate, purely on population genetics grounds. A naive reason for the innovation probability to decrease would be that the pool of total available domain shapes is small, which would affect the innovations at increasing size, while duplications are free of this constraint. However, this would imply that the currently observed genomes are already at the limit of their capabilities in terms of producing new protein shapes, while the current knowledge of protein folding does not seem to indicate this fact [3,35]. On the other hand, this argument could hold on effective grounds, because of the action of other constraints. For example, supposing that gain of new domains in a genome is often originated by horizontal transfer or by mutation from prior domains, not all domains are equally likely to appear: those that are rare are less likely to be new introductions either because horizontal transfers involving them will be rare, or because the mutational bridge from their precursors is very long. This aspect is partially covered by the specific variant of the CRP, which has the best agreement with the data. Also, the limited availability of domain classes could be true within a certain environment, where the total pool of domain families is restricted. We cannot exclude that the same kind of bias could be due to technical problems in the recognition and classification of new shapes in the process of producing the data on structural domains. If recognition algorithms tend to project shapes that are distinct from known ones, they could classify new shapes as old ones with a rate that increases with proteome size, leading to the observed scaling.

### Possible computational cost of domain addition

Finally, another reason for *p*_*N *_to decrease could be selective. New domains are only likely to be selected if they perform a biological function that is not covered by pre-existing domains or their combinations. Hence, as the number of domains increases, the chance a new one will be accepted should decrease. Along similar lines, we would like to suggest that a reason for *p*_*N *_to decrease with *n *could be related not only to function, but also to the cost for 'wiring' new domains into existing interaction networks. The argument is related to the so-called 'complexity hypothesis' for horizontal transfers [36-39], which roughly states that the facility for a transferred gene to be incorporated depends on its position and status in the regulatory networks of the cell. We suppose that, given a genome with *n *domains (or for simplicity monodomain genes) and *F *domain families, the process leading to the acceptance of a new domain family, and thus to a new class of functions, will need a re-adaptation of the population of all the domain families causing an increase *δn *in the number of genes. This increase is due to an underlying optimization problem that has to adapt the new functions exploited by the acquired family to the existing ones (by rewiring and expanding different interaction networks). To state it another way, we imagine that in order to add *δF *new domain classes, or 'functions', it is necessary to insert *δn *new degrees of freedom ('genes') to be able to dispose of the functions. Now, generically, the computational cost for this optimization problem (which, conceptually, may be regarded as a measure of the evolvability of the system) could be a constant function of the size (and thus *δn *~ *δF*), or else polynomial or exponential in *F *(that is, *δn *~ *F*^*d *^*δF*, where *d *is some positive exponent, or *δn *~ exp(*F*)*δF*, respectively). Integrating these relations gives *n *~ *F *in the first case, *n *~ *F*^*d*+1 ^in the second, and *n *~ exp(*F*) in the third. Inverting these expressions shows that the first choice leads to the linear scaling of the model of Gerstein and coworkers, while the second two correspond to the CRP, and to a sublinear *F*(*n*), which could follow a power law or logarithmic, depending on the computational cost. In other words, following this argument, accepting a new domain family becomes less likely with increasing number of already available domain families, as a consequence of a global constraint. This constraint comes from the trade-off between the advantage of incorporating new functions and the energetic or computational cost to govern them (both of which are related to selective pressure). This hypothesis could be tested by evaluating the rates of horizontal trasfers carrying new domain classes in an extensive phylogenetic analysis.

## Conclusion

The model and data together indicate that evolution acts conservatively on domain families, and shows increasing preference with genome size to exploiting available topologies rather than adding new ones. A final point can be made regarding the number of observed domains. The model assumes that the new domain classes are drawn from an infinite family of topologies, which can be even continuous [29], and leads to a discrete and small number of classes at the relevant sizes. Although physical considerations point to the existence of a small 'menu' of three-dimensional shapes available to proteins [40], the validity of our model would imply that the empirical observation of a small number of folds in nature does not count as evidence for this thermodynamic property of proteins, but may have been a simple consequence of evolution.

## Materials and methods

### Data

We considered data on protein domains on 327 bacteria, 75 eukaryotes, and 27 archaea from the SUPERFAMILY database [19].

### Model and simulations

The quantitative duplication-innovation-loss evolutionary models were explored by mean-field theory and direct simulation.

## Abbreviations

CRP: Chinese restaurant process.

## Authors' contributions

MCL designed and performed research, and wrote the paper. BB designed and performed research. AS and PH performed research. All authors read and approved the final manuscript.

## Additional data files

The following additional data are available with the online version of this paper. Additional data file 1 contains supplementary information on the model and data analysis.

## Supplementary Material

Additional File 1 Supplementary information on the model and data analysis. Click here for file
