# Supplementary material for: Universal features in the genome-level evolution of protein domains
Source: Genome Biol. 2009 Jan 30;10(1):R12. doi: 10.1186/gb-2009-10-1-r12 (PMC2687789; doi:10.1186/gb-2009-10-1-r12)
Supplement: Additional File 1 — Supplementary information on the model and data analysis. [file gb-2009-10-1-r12-S1.pdf]

Additional Files

ADDITIONAL DOCUMENTATION

A1. CUMULATIVE DISTRIBUTIONS FOR THE INTERNAL USAGE OF DOMAINS

This section briefly discusses the cumulative histograms of domain usage for data and models. Figure A1.1 confirms the markedly power-law behavior observed for the histograms and predicted by the model. Comparison with the predictions of the CRP model (figure A1.2) shows faster decay for $\alpha = 0$. While in good agreement with the observed number of domain classes with increasing size (figure 1B), this parameter choice is unsatisfactory on the quantitative side for the domain distribution in classes. This feature, already visible in figure 2B of the main text, is even more marked from the cumulative histograms. Better-fitting values are in the range $\alpha = 0.5 - 0.7$. The CRP with specific domain classes (figure A1.3) has the same qualitative behavior as the standard model for the distributions, while fitting well the scaling of the classes of higher values of α (figure 1B and section A4 below).

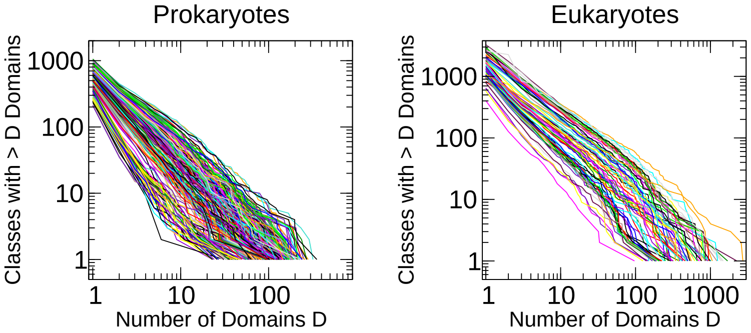

Additional Figure A1.1 Empirical cumulative distributions of domain usage for domain classes of the SUPERFAMILY database. The x-axis reports domain class sizes in number of domains D while the y-axis refers to the histogram of the number of domain classes containing more than D domains. The left panel is based on the same data on the 327 prokaryotes of figure 2A in the main text. The right panel refers to the 75 eukaryotes in the data set. The genome sizes are not color-coded to show individual plots.

A2. RESULTS FOR FOLD DOMAIN CLASSES

All data shown in the main text refer to the superfamily taxonomy level, and come from the SUPERFAMILY database. In this section, we report the results of the same analysis in terms of SCOP folds, which show that this category has essentially the same behavior as the previous one (figure A2.4). While by definition there are more superfamilies than folds, the number of domain classes versus genome size has very similar scaling in the two cases. The two plots collapse almost exactly, when folds are rescaled by the

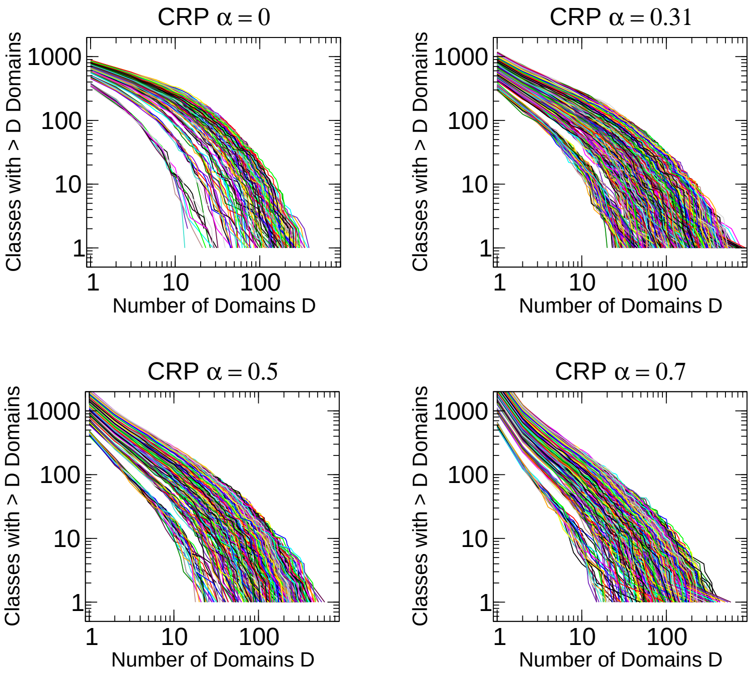

Additional Figure A1.2 Cumulative histograms of domain usage for 50 realizations of the CRP at genome sizes between 500 and 8000. Increasing values of α are plotted in lexicographic order.

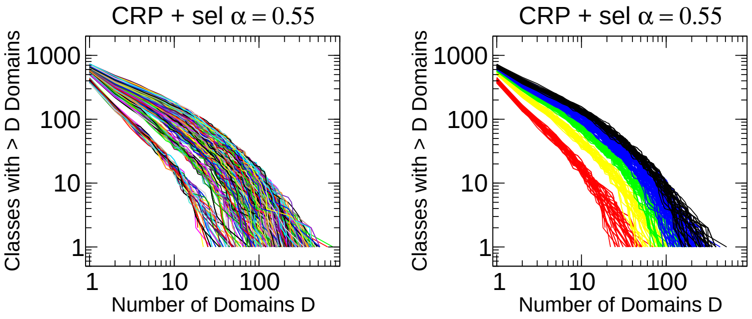

Additional Figure A1.3 Cumulative histograms of domain usage for 100 realizations of the CRP with specific classes at genome sizes between 1000 and 8000. In this size range the model variant produces essentially identical distributions to the conventional CRP, with better agreement on the growth in terms of domain classes (see section A4). The left panel is color-coded as figure 2B of the main text.

ratio (1443/884) of superfamilies per folds (A2.5). Furthermore, power-law fits of the experimental data for prokaryotes yield an exponent α between 0.3 and 0.4 for both categories, and logarithmic fits are also in agreement.

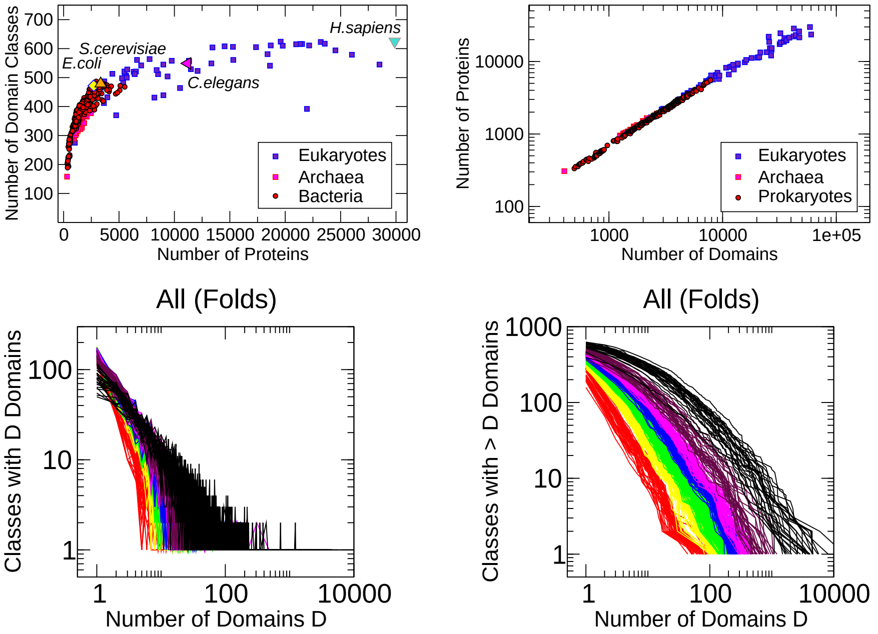

Additional Figure A2.4 Top: Number of fold classes versus genome size measured by number of proteins and as number of distinct domains. The plot in the left panel is equivalent to figure 1A, except that the x-axis reports number of proteins scored in the genome, rather than genome size in domains. Since these two quantities are quite markedly linearly related (right panel), using either parameter does not affect the observed trends. Bottom: histogram (left panel) and cumulative histogram (right panel) of domain classes for all genomes in the data set (eukaryotes, prokaryotes and archaea).

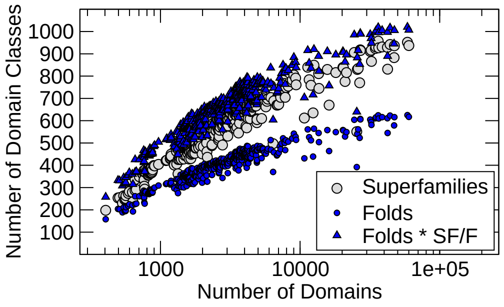

Additional Figure A2.5 Comparison of the scaling of folds and superfamilies plot as a function of genome size. The plots refer to all genomes in the SUPERFAMILY database. The plot for folds (blue small circles) overlaps quite well with the plot for superfamily (large grey circles) when multiplied by the ratio of the total number of domain classes in the two taxonomies (1443/884).

A3. DOMAIN CLASSES VERSUS GENOME SIZE IN EUKARYOTES, LONGEST TRANSCRIPT PER GENE.

The most recent version (1.73) of the SUPERFAMILY genome assignments, provides domain associations by scoring the longest transcript per gene. Figure A3.6 reports the behavior of the number of domain

classes in these data. While proteome sizes are overestimated roughly by a factor of two due to alternative splicing, the collective behavior and the sublinear trend of $F(n)$ do not change.

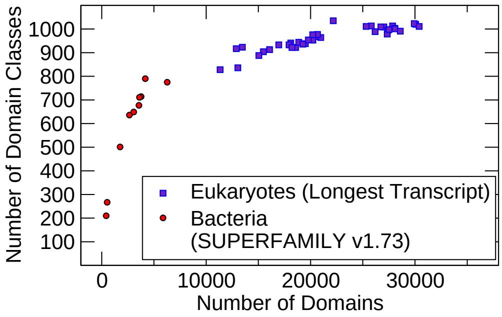

Additional Figure A3.6 Plot of the number of domain classes versus genome size, for eukaryotic genomes where scored sequences correspond to the longest non-overlapping transcript per gene. This avoids double counting of domains due to splicing variants of the same protein with overlapping sequences.

A4. CRP MODEL WITH SPECIFIC DOMAIN CLASSES AND ANALITYCAL MEAN FIELD EQUATIONS

In this section we discuss the variant of the CRP model introduced in the main text and its analytical treatment. We first give some more details on the definition of the model. Generically, we consider the following genetic algorithm. For each genome size n, the configuration is a set of M genomes $\{g_1(n), ..., g_M(n)\}$, where each genome is a set of D domain classes populated by some domains. An iteration is divided into two steps. A first "proliferation" step generates qM genomes, where q is a positive integer, $\{g_1^{'}(n), ..., g_{qM}^{'}(n)\}$, using the standard CRP move. A second "selection" step discards the $(q-1)M$ individuals with higher cost.

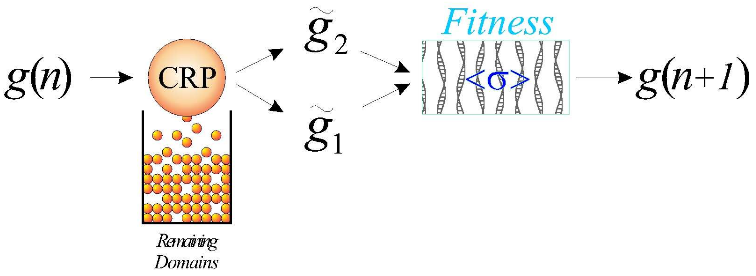

Additional Figure A4.7 Scheme of the CRP variant with domain specificity. At size n, multiple (two in the figure) "virtual" moves are generated with a standard CRP model, at fixed parameters. Subsequently, the moves with lowest cost (one in this case) are selected. In our case, the cost function is chosen by comparing the domain usage of the model genome with the empirical usage of specific domain families

The cost function, for a generic model genome g, can be a function $\mathcal{F}(g)$, that takes into account some phenomenological features observed in the data. We choose to include in $\mathcal{F}$ a minimal amount of empirical

information on the occurrence of each domain class contained in figure 1C. In other words, we distinguish between "universal" domain classes, used in most of the genomes, and "contextual" ones, occurring only in a few examples. As discussed in the main text, this is sufficient to obtain quantitative agreement with the observed domain distributions (figures 1B and 2B), which are not given to the model as an input. If domain classes are indexed by $i = 1..D$ ($D = 1443$ for Superfamilies), we define the variable σ_i^g as follows

$$\sigma_i^l = \begin{cases} 1 & \text{if domain class } i \text{ is present in genome } g \\ -1 & \text{if domain class } i \text{ is absent in genome } g \end{cases} .$$

The cost function of that genome is then defined as

$$\mathcal{F}(g) = \exp\left(\sum_{i=1}^{D} \sigma_i^g \langle \sigma_i^{\mathrm{EMP}} \rangle\right) \quad ,$$

where $\langle \sigma_i^{\mathrm{EMP}} \rangle$ is the empirical average of the same observable:

$$\langle \sigma_i^{\mathrm{EMP}} \rangle = \frac{1}{G} \sum_{g=1}^{G} \sigma_i^{g,\mathrm{EMP}} \quad .$$

In the above formula G is the number of observed genomes in the data set. For example, in the case of prokaryotes in the SUPERFAMILY database, $G = 327$ and, calling Ξ_i the function plotted in figure 1C, we have simply $\langle \sigma_i^{\mathrm{EMP}} \rangle = 2\Xi - 1$.

For the analitycal treatment, we considered the case $M = 1$, $q = 2$, where at each iteration, one genome is selected from a population of two. Starting from configuration $g(n)$, in the proliferation step genomes $g^{'}, g^{''}$ are generated with CRP rules, and the selection step chooses $g(n+1) = \mathrm{argmax}(\mathcal{F}(g^{'}), \mathcal{F}(g^{''}))$. In this case, since the selection rule chooses strictly the maximum, it is able to distinguish the sign of $\langle \sigma_i^{\mathrm{EMP}} \rangle$ only. For this reason, it is sufficient to account for the positivity (which we label by "+") and negativity ("-") of this function for a given domain index i. Note that this reduces the effective parameters to one only: the fraction of universal domain classes. The genomes $g^{'}$ and $g^{''}$ proposed by the CRP proliferation step can have the same (labeled by "1"), lower ("1_+") or higher ("1_-") cost than their parent, depending on p_O, p_N and by the probabilities to draw a universal or contextual domain family, p_+ and p_- respectively. Using these labels, the scheme of the possible states and their outcome in the selection step is given by the table below.

proliferation $(g^{'}, g^{''})$	probability	selection
$(1, 1)$	p_O^2	old
$(1, 1_-)$	$2\, p_O\, p_N\, p_-$	old
$(1, 1_+)$	$2\, p_O\, p_N\, p_+$	new+
$(1_+, 1_+)$	$p_N^2\, p_+^2)$	new+
$(1_+, 1_-)$	$2\, p_N^2\, p_-\, p_+$	new+
$(1_-, 1_-)$	$p_N^2\, p_-^2$	new−

From this table, it is straightforward to derive the modified probabilities $\hat{p}_O$ and $\hat{p}_N$ of the complete iteration:

$$\hat{p}_O = p_O\,(p_O + 2\,p_N p_-)$$

$$\hat{p}_N = p_N\,(p_N + 2\,p_O p_+) = p_{N+} + p_{N-} \quad ,$$

where $p_{N+} = p_N p_+ (2 - p_N p_+)$ and $p_{N-} = p_N^2 (1 - p_+)^2$ are the probabilities that the new domain is drawn from the universal or contextual families respectively.

We now write the macroscopic evolution equation for the number of domain families using the same procedure as in the main text. Calling $k^+(n)$ and $k^-(n)$ the number of domain classes that have positive or negative $\langle \sigma_i^{\mathrm{EMP}} \rangle$ and are *not* represented in $g(n)$,

$$\begin{cases} \partial_n F(n) = & \hat{p}_N \\ \partial_n k^+(n) = & -\hat{p}_{N+} \\ \partial_n k^-(n) = & -\hat{p}_{N-} \end{cases} .$$

Now, $p_+ = k^+/(k^- + k^+) = k^+/(D - F(n)$, so that we can rewrite

$$\begin{cases} \partial_n F(n) = & (\frac{\alpha F(n)+\theta}{n+\theta}) \left[\frac{\alpha F(n)+\theta}{n+\theta} + \frac{2k^+(n)}{D-F(n)} (\frac{n-\alpha F(n)}{n+\theta}) \right] \\ \partial_n k^+(n) = & -(\frac{\alpha F(n)+\theta}{n+\theta})\, \frac{k^+(t)}{D-F(n)} \left[2 - (\frac{\alpha F(n)+\theta}{n+\theta}) \frac{k^+(n)}{D-F(n)} \right] \\ \partial_n k^-(n) = & -(\frac{\alpha F(n)+\theta}{n+\theta})^2\, (\frac{k^+(n)}{D-F(n)})^2 \end{cases} \tag{1}$$

The above equations have the following consistency properties

- $\partial_n \Big(k^+ + k^- + F \Big) = 0$, hence $k^+ + k^- + F = D \quad \forall n$.
- $\partial_n F \leq 1$, hence $F(n) \leq n$.

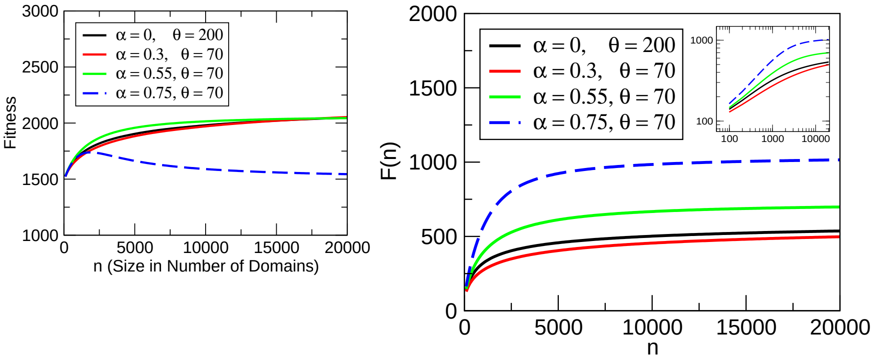

Additional Figure A4.8 Numerical solutions of the mean-field equations of the CRP model with selection of specific domain classes. Left panel: cost function $\mathcal{F}(n)$ for different values of α. Right panel: F(n) plotted in linear and logarithmic (inset) scales.

- $\partial_n F \geq 0$, $\partial_n k^+ \geq 0$ and $\partial_n (F + k^+) \geq 0$ so that F grows faster than k^+ decreases.

Choosing the initial conditions from empirical data $n_0, F(n_0)$ size and number of domain classes of the smallest genome, we have, since $F(n_0) < n_0$ and $\alpha \leq 1$,

$$\frac{\alpha F(n_0) + \theta}{n_0 + \theta} < 1 \ .$$

It is simple to verify that under this condition the system always has solutions that relax to a finite value $F_\infty < D$. Indeed, after the time n^* where $k^+(n*) = 0$, the equations reduce to $\partial_n k^+ = 0$, $k^- = D - F$ and

$$\partial_n F(n) = \left(\frac{\alpha F(n) + \theta}{n + \theta}\right)^2$$

immediately giving our result.

Numerical solutions of Eq. (1) give the same behavior for $F(n)$ as the direct simulations (figures A4.8A, and figure 1B of the main text). In particular, while this function grows as a power law for small genome sizes, it saturates at the relevant scale, giving good agreement with the data. This behavior is connected to the finite size of the pool of universal domain families, which we can interpret as the effect of a certain optimality in the core functions of the different organisms. The internal laws of domain usage of this model were obtained from direct simulations only, and, as discussed in the main text, give a more quantitative agreement with the data (figure 2B of the main text). Finally, one interesting point can be made about the dynamics of the cost function. Figure A4.8B, shows that, for large values of α (above 0.7) this function reaches a maximum at sizes between 2000 and 4000. This is also where most of the genomes in the data set are found, indicating that this range of genome sizes may allow the optimal usage of universal and contextual domain families.

A5. OTHER VARIANTS OF THE CRP

We discuss here mean-field arguments for the robustness of our results on the asymptotics of $F(n)$ for two variants of the original model, including a small domain loss rate and global duplications.

a. Global Duplications. One can consider the presence of global duplication moves. At each time step, if duplication is chosen, a number of domains selected with $q > 1$ trials from a binomial distribution with parameter p^i_O is duplicated in the same time step. The innovation step remains the same. In this case, it is not possible to measure time with the size n of the genome, but this observable follows the evolution equation

$$\dot{n} = qp_O + p_N \ , \tag{2}$$

where $\dot{}$ indicates the derivative with respect to time t. In terms of t, our mean field equations are worked out simply as $\dot{F}(t) = p_N$ and $\dot{K}_i(t) = qp^i_O$. Using Eq. (2), they can be simply converted in terms of n, yielding

$$\partial_n F(n) = \frac{\alpha F(n) + \theta}{qn + (q-1)\alpha F(n) + \theta} \ ,$$

and

$$\partial_n K_i(n) = \frac{K_i - \alpha}{n + \frac{\theta}{q}} \ .$$

The first equation gives as leading scaling $F(n) \sim n^{(\alpha/q)}$, showing that the growth of F is pushed towards effectively lower values of α by global duplications, as a consequence of the rescaling of time by the global moves. The dynamics for K_i, instead, is affected only by a renormalization of the parameter θ. The qualitative results of the model are therefore stable to the introduction of a global duplication rate, in the hypothesis that the extent of these duplications does not scale with n.

b. Domain Loss. A second interesting variant of the model considers the introduction of a homogeneous domain deletion, or loss rate. Domain loss is known to occur in genomes. However, it is not considered in our basic model for simplicity and economy of parameters. In order to introduce it in the CRP, we define a loss probability $p_L = \delta$. This is equally distributed among domains, so that the *per class* loss probability is $p^i_L = \delta \frac{K_i}{n}$. Consequently, the duplication and innovation probability p_O and p_N are rescaled by a factor $(1 - \delta)$. The mean-field evolution equation for the number of domain classes becomes

$$\dot{F}(t) = (1-\delta)\frac{\alpha F + \theta}{n + \theta} - \delta \frac{F(1,n)}{n} \ ,$$

where the sink term for F derives from domain loss in classes with a single element, quantified by $F(1, n)$.

In order to solve this equation, one needs an expression for $F(1, n)$. Here, we report an argument based on the fact that in direct simulation of the model, for large n, $F(1, n) = \gamma F(n)$, with $0 < \gamma < 1$ (data not shown). This trend is also confirmed by the empirical data. Using this experimentally motivated ansatz, we can show that for small δ, the scaling of $F(n)$ is subject only to a small correction. In the model including domain deletions, more genomes of the same history can have the same size. Again, since model time t (which should be regarded as a fictitious variable, with a complex relation with evolutionary time in generations) does not correspond genome size, one has to consider the evolution of n with t, given in this model simply by $\dot{n} = 1 - 2\delta$. Using this equation it is possible to obtain the evolution equation for $F(n)$. Considering an expansion in small δ and large n, this reads to first order

$$\frac{\partial_n F(n)}{F(n)} = \frac{\alpha}{n} \left[1 + \delta \left(\frac{\alpha - \gamma}{\alpha} \right) \right] \quad .$$

The above equation gives the conventional scaling for $F(n)$, with the aforementioned correction. Note that the correction could be positive or negative, depending on the relative values of α and γ. An analogous argument holds for $\alpha = 0$.
